# Can the exposome help solve the alcohol and cancer mystery?

**DOI:** 10.1371/journal.pbio.3004100

**Published:** 2026-11-05

**Authors:** Vasilis Vasiliou, Arthur Stem, David C. Thompson

**Affiliations:** Department of Environmental Health Sciences, Yale School of Public Health, Yale University, New Haven, Connecticut, United States of America

## Abstract

Alcohol consumption is a preventable driver of many cancers, yet a central biological question remains unresolved: by what mechanisms does ethanol promote malignancy? We propose that alcohol in the context of the **exposome** (i.e., the network of chemical, biological, and social determinants that interact with ethanol across the life span) provides a unifying framework for understanding alcohol-related carcinogenesis. Viewed through the lens of the exposome, alcohol is not only as a direct carcinogen but also as a systemic amplifier of environmental risk acting across multiple levels of biological organization, from molecular damage to whole-body physiology. This reframing has important implications for mechanistic research, precision prevention, and public-health policy.

## Abstract 2

Alcohol is a preventable cause of cancer, but the mechanisms linking its consumption to malignancy remain incompletely understood. This Unsolved Mystery explores alcohol’s carcinogenic effects through an exposome lens, arguing that alcohol acts as an amplifier of environmental risk, with important implications for prevention, public health policy, and future research.

## Introduction

Alcohol consumption has long been associated with increased cancer risk, and yet a central question remains: why does similar exposure result in markedly different outcomes across individuals and populations? Epidemiological studies have consistently linked alcohol to a variety of cancers, but the magnitude and tissue-specificity of these associations vary widely [1]. The US National Academies of Sciences, Engineering, and Medicine recently conducted a comprehensive review of the epidemiological evidence associating alcohol consumption to cancer [2]. While the report acknowledged a growing body of studies supporting an association, it also highlighted important limitations, including residual confounding, inconsistent dose–response relationships, and variability across populations and cancer types. Residual confounding is particularly difficult to eliminate because alcohol consumption frequently co-occurs with other risk factors, such as tobacco use, dietary patterns, environmental exposures, and disparities in healthcare access [3]. While these challenges do not negate the association between alcohol and cancer, they underscore a deeper scientific problem: understanding precisely how alcohol promotes carcinogenesis within the complex biological and environmental contexts in which it is consumed [4].

Cancer development reflects the balance between processes that promote or suppress malignant transformation. These include inherited genetic variation, genomic integrity, epigenetic state, and immune surveillance, which together govern cell proliferation and death; the latter three are themselves shaped by the exposome, the cumulative external exposures that include environmental, dietary, and social exposures accumulated across the life span. Studying alcohol in the context of the exposome addresses a specific and persistent gap: why individuals with comparable ethanol intake differ so markedly in cancer risk, latency, and tumor site.

The mechanistic case spans three levels of biological organization. At the molecular level, the evidence is well established: ethanol disrupts redox homeostasis, damages DNA, impairs mitochondrial function, and generates reactive metabolites that modify macromolecules. At the tissue level, it compromises epithelial barriers, promotes inflammation, and alters hormonal and metabolic regulation. Interactions with co-occurring exposures are the least characterized and, we argue, the most consequential. Alcohol and tobacco smoke combine more than additively in the upper aerodigestive tract, and the shared role of nitrosamines and acetaldehyde provides a plausible mechanistic basis for this synergy. Whether comparable interactions occur with persistent environmental contaminants such as per- and polyfluoroalkyl substances (PFAS) and pesticides remains largely untested, despite overlapping targets in hepatic metabolism, redox regulation, and endocrine signaling. Advances in exposomic technologies, including high-resolution mass spectrometry and integrated multi-omics, now make these interactions tractable.

In this Unsolved Mystery, we investigate the various mechanisms by which alcohol interacts with exposome and affects cancer, asking the question of whether a greater understanding of this interaction will help us to answer key questions around why alcohol use results in so many different outcomes across different individuals.

## Why does alcohol’s carcinogenic risk vary so widely?

Alcohol consumption has long been portrayed as a modifiable cancer risk factor [5], with some epidemiological analyses estimating that ≈5% of new cancer cases in the United States are attributable to alcohol use [6]. The International Agency for Research on Cancer classifies alcoholic beverages, ethanol, and acetaldehyde associated with the consumption of alcoholic beverages as Group 1 human carcinogens, a position based largely on epidemiological associations [7]. Numerous cohort and case–control studies have reported positive dose-dependent associations between alcohol intake and cancers of the breast, liver, colorectum, oral cavity, pharynx, larynx, and esophagus. In some cases, risk increases with higher levels of alcohol consumption, particularly when alcohol use occurs in combination with tobacco smoking. However, the magnitude and consistency of these associations vary widely across populations, study designs, and cancer types [8–11], making the establishment of clearly defined risk thresholds more challenging. Nevertheless, it is important to recognize that there is no safe level of alcohol consumption with respect to cancer risk.

The biological consequences of alcohol consumption are also highly context-dependent, being influenced by factors such as dose, co-exposures, and genetic susceptibility. In hepatocellular carcinoma (HCC), sustained ethanol intake promotes liver injury, steatohepatitis, fibrosis, and cirrhosis—chronic pathological states that establish a pro-carcinogenic microenvironment [10]. Chronic infection with hepatitis B or C virus is an especially important co-factor in this process. Viral hepatitis independently drives hepatic inflammation and fibrosis, and alcohol consumption can synergistically increase HCC risk in individuals with chronic viral hepatitis compared with either exposure alone [12]. Notwithstanding this scenario, HCC occurs only in one of every 50 heavy alcohol consumers. In colorectal cancer, alcohol exposure has been linked to disruption of one-carbon metabolism, acetaldehyde-induced DNA damage, gut microbiota dysbiosis, and immune dysregulation [13]. Even so, these mechanistic pathways do not fully explain the variability observed in human populations.

Genetic susceptibility further complicates the picture. Allelic variants in aldehyde dehydrogenase 2 (ALDH2) that impair the detoxification of acetaldehyde (e.g., ALDH2*2) are common in East Asian populations, and are associated with substantially increased risks of esophageal cancer among individuals who consume alcohol [14]. Evidence also suggests that gene–diet interactions, metabolic differences, and hormonal factors might modify alcohol-related cancer risk, highlighting the importance of considering the broader biological context in which alcohol exposure occurs [15,16].

The burden of alcohol-associated cancers is also unevenly distributed across populations and environments. Differences in dietary patterns, metabolic health, patterns of alcohol consumption, and co-occurring environmental exposures contribute to substantial regional variation in cancer risk. In many Western populations, alcohol consumption contributes to breast cancer incidence, reflecting interactions between ethanol metabolism and hormone signaling [17]. Socioeconomic conditions further shape these patterns, as alcohol use frequently co-occurs with environmental pollution, dietary imbalances, psychosocial stress, and/or unequal access to healthcare [18]. These observations suggest that alcohol-related cancer risk can be strongly influenced by biological and environmental contexts [19]. These observations also point to a limitation in how alcohol is often studied. Epidemiological analyses routinely examine alcohol alongside correlated factors (e.g., smoking, socioeconomic status, and genetic variation) but are unlikely to capture the full network of interacting chemical and biological co-exposures [19,20]. These constraints highlight a key challenge in the field: understanding how alcohol promotes carcinogenesis within the complex biological and environmental contexts in which it is consumed. Its biological effects manifest within a network of interacting exposures that have the potential to influence both susceptibility and cancer progression.

## Can the exposome help solve the alcohol–cancer puzzle?

The exposome, the total exposures of an individual over a life span, provides a conceptual framework for understanding this complexity. First proposed as a complement to the genome, the exposome encompasses the totality of environmental exposures experienced throughout the life span (including chemical agents, diet, lifestyle, radiation, the gut microbiota, and psychosocial factors), as well as the internal biochemical responses these exposures generate [21]. Within this framework, alcohol emerges not simply as an isolated toxicant but as a biochemical interface that has the potential to interact with numerous environmental and biological factors. For example, persistent pollutants (such as PFAS, pesticides, and heavy metals) share metabolic and redox pathways with ethanol [22,23]. As discussed above, social determinants, (such as diet, psychosocial stress, and patterns of alcohol consumption) can modify biological responses to alcohol exposure [24]. Viewing alcohol through the exposome, therefore, reframes the scientific question. Rather than asking simply what ethanol does, we should instead ask **how ethanol interacts with the broader landscape of exposures that shape human biology and cancer risk.**

### How can alcohol consumption damage DNA and cellular defenses?

At the molecular level, ethanol metabolism initiates a cascade of biochemical events that can compromise genomic stability and promote cancer (Fig 1). Following ingestion, ethanol is oxidized primarily by alcohol dehydrogenase (ADH) to acetaldehyde, the first and most reactive metabolite of ethanol. Under normal conditions, acetaldehyde is rapidly detoxified to acetate by ALDH2. However, under chronic or heavy alcohol consumption, additional metabolic pathways become involved. In particular, cytochrome P450 2E1 (CYP2E1) is induced and contributes to acetaldehyde production and the generation of reactive oxygen species (ROS) [13,25].

**Fig 1:**
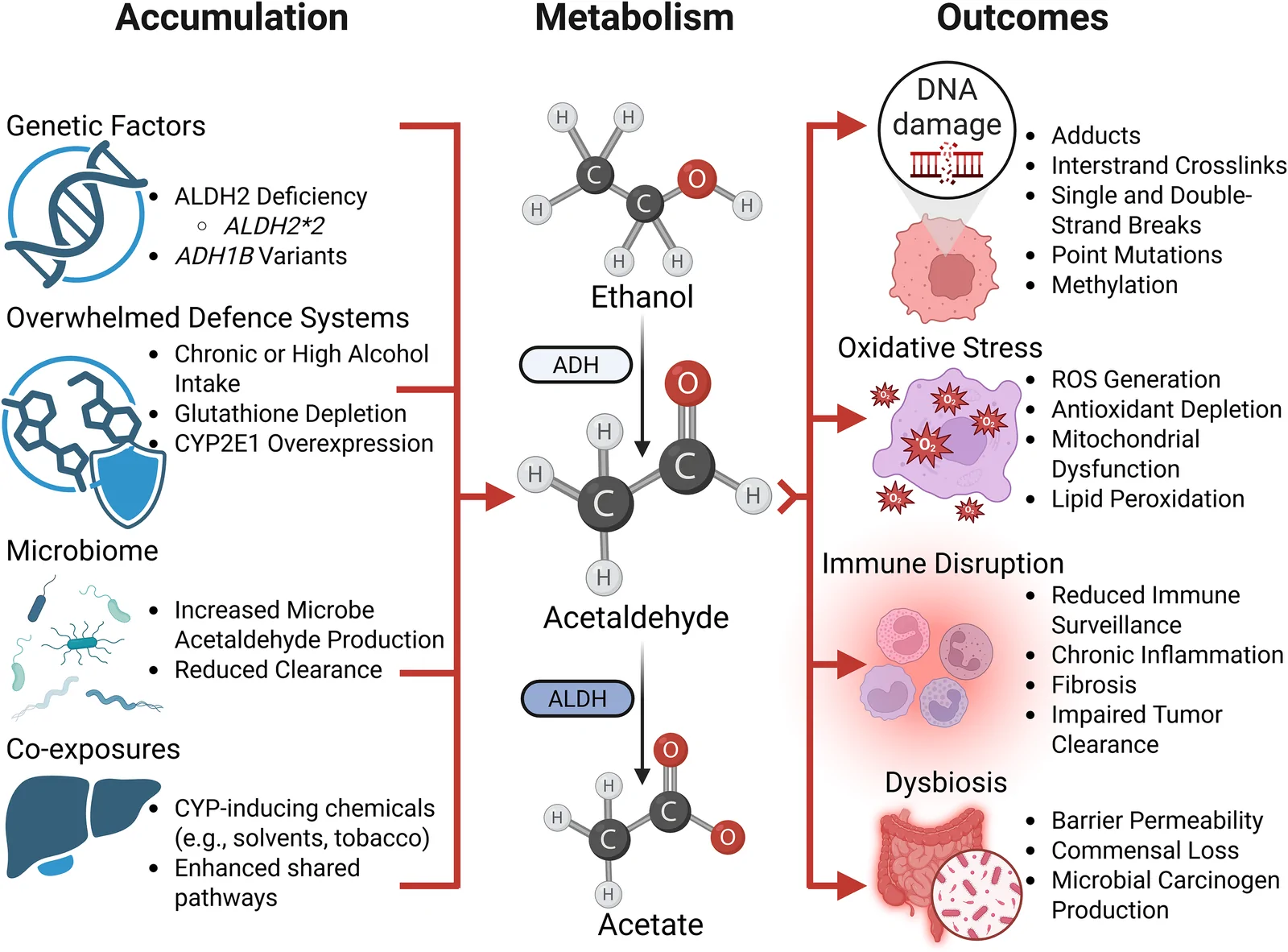
Host and environmental determinants of acetaldehyde accumulation and cancer risk. Ethanol is metabolized to acetaldehyde by alcohol dehydrogenase (ADH), and then on to acetate by aldehyde dehydrogenase (ALDH). Acetaldehyde is a reactive intermediate, the accumulation of which can be influenced by host genetic variation (e.g., ALDH2*2, ADH*1B*), overwhelmed detoxification systems (e.g., glutathione depletion, CYP2E1 induction), microbiota-derived production, and co-exposures that induce xenobiotic metabolism. These factors collectively increase acetaldehyde burden and drive multiple carcinogenic processes, including DNA damage (e.g., adduct formation, strand breaks, and altered methylation), oxidative stress (e.g., ROS generation, mitochondrial dysfunction, lipid peroxidation), immune dysregulation (e.g., chronic inflammation, impaired tumor surveillance), and gastrointestinal dysbiosis (e.g., increased barrier permeability and microbial carcinogen production). CYP2E1, cytochrome P450 2E1; ROS, reactive oxygen species. Created with Biorender.com.

Acetaldehyde is a highly reactive electrophile capable of forming covalent adducts with DNA and proteins (Fig 2) [26]. These interactions can produce DNA interstrand crosslinks, point mutations, and characteristic lesions, such as N^2^-ethylidene-deoxyguanosine adducts [26]. Such damage interferes with DNA replication and transcription fidelity, and can initiate mutagenic processes that contribute to carcinogenesis [27,28]. By increasing intracellular acetaldehyde levels, chronic ethanol consumption can, therefore, promote genotoxic injury.

**Fig 2:**
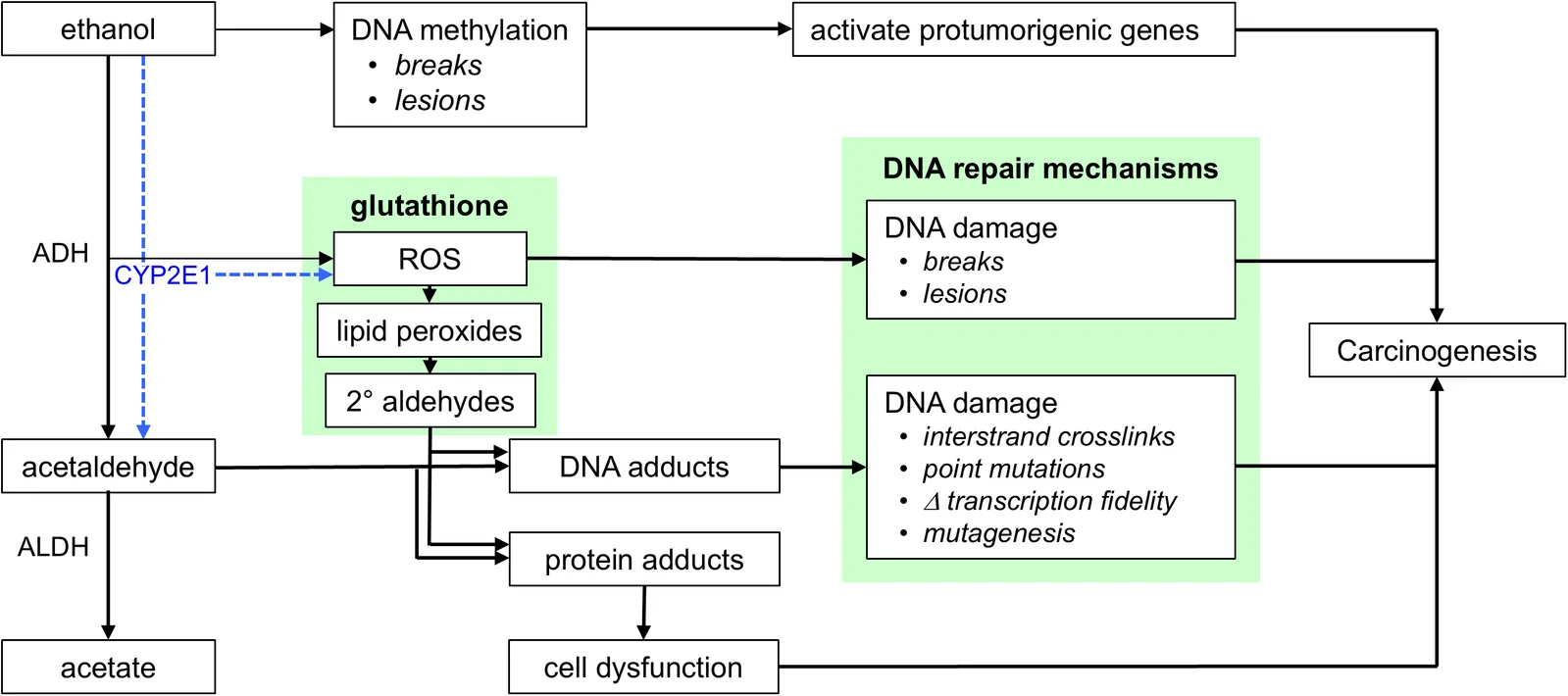
Genotoxic and epigenetic mechanisms linking ethanol metabolism to carcinogenesis. Ethanol metabolism results in acetaldehyde accumulation and CYP2E1-derived reactive oxygen species (ROS). These species drive lipid peroxidation and secondary (2°) reactive aldehyde production, processes normally constrained by glutathione, which becomes depleted under chronic alcohol exposure. Acetaldehyde and ROS-derived species converge on DNA and proteins, forming adducts that impair cellular function and induce a spectrum of genotoxic lesions, including strand breaks, interstrand crosslinks, point mutations, and reduced transcriptional fidelity. Ethanol exposure also disrupts epigenetic regulation through aberrant DNA methylation, promoting the activation of pro-tumorigenic genes. When these genotoxic and epigenetic insults exceed DNA repair capacity, genomic instability ensues, ultimately driving carcinogenesis. ADH, alcohol dehydrogenase; ALDH, aldehyde dehydrogenase; CYP2E1, cytochrome P450 2E1; Δ, change in.

The likelihood of genotoxic effects of acetaldehyde in an individual can be influenced by genetic polymorphisms that affect the synthesis or metabolism of acetaldehyde and/or alter DNA repair pathways. For example, inactivating variants in *ALDH2* (e.g., ALDH2*2 present in ≈40% of the population in East Asian countries) can lead to reduced acetaldehyde clearance, resulting in the accumulation of acetaldehyde after ethanol consumption [29]. Notably, these individuals exhibit a markedly increased risk of esophageal squamous cell carcinoma, especially when alcohol consumption is combined with tobacco smoking [14]. In addition, variants in *ADH1B* that increase the rate of ethanol oxidation to acetaldehyde can increase acetaldehyde exposure and alcohol-related cancer risk. Genome-wide association studies most robustly implicate *ALDH2* variants (particularly for upper aerodigestive tract cancers) with more limited evidence for *ADH1B* and for other cancer sites, highlighting the importance of gene–environment interactions [30–32].

Cells possess multiple DNA repair systems that counteract the genotoxic effects of acetaldehyde and help maintain genomic stability. These include the Fanconi anemia pathway, base excision repair, nucleotide excision repair, mismatch repair, and homologous recombination. The Fanconi anemia pathway holds a central role in resolving acetaldehyde-induced DNA interstrand crosslinks, one of the most toxic forms of DNA damage [33]. Recent studies have demonstrated that mice deficient in both ALDH2 and FANCD2 (a key component of the Fanconi anemia DNA interstrand crosslink repair pathway) accumulate high levels of DNA damage and exhibit increased mutation burdens in hematopoietic stem cells, highlighting the importance of coordinated aldehyde detoxification and DNA repair [34]. Consistent with these findings, FANCD2 facilitates the repair of acetaldehyde-induced crosslinks in epithelial tissues (such as oral keratinocytes), and in vitro studies indicate that the Fanconi anemia pathway also participates in resolving acetaldehyde-induced DNA crosslink structures [35]. In addition to acetaldehyde-derived DNA adducts, ethanol metabolism generates ROS, particularly via CYP2E1 activity, and perturbs cellular redox homeostasis through increased NADH production during alcohol and acetaldehyde oxidation, which can impair mitochondrial function and promote oxidative stress [36]. These ROS induce oxidative DNA lesions that are primarily repaired through base excision repair. Homologous recombination can repair double-strand DNA breaks that arise from replication stress and oxidative damage [37–39]. Mismatch repair pathways can also influence cellular responses to acetaldehyde exposure. For example, combined disruption of aldehyde metabolism and mismatch repair components has been associated with increased tumor formation in experimental animal models [40]. A role for nucleotide excision repair in removing acetaldehyde-induced DNA lesions has been demonstrated in cells deficient in proteins, such as XPA, which exhibit a reduced capacity to repair acetaldehyde-damaged DNA [41]. Within an exposome framework, variability in DNA repair capacity becomes particularly important. Genetic variation in DNA repair pathways can modify the cellular response to environmental exposures (including alcohol, tobacco smoke, dietary aldehydes, and pollutants), thereby influencing the cumulative burden of DNA damage experienced by cells.

Ethanol metabolism adversely affects cellular redox balance [42]. Oxidation of ethanol increases intracellular NADH levels, altering the NAD^+^/NADH ratio and impairing mitochondrial respiration [36]. As noted, chronic alcohol consumption induces CYP2E1, which metabolizes ethanol while generating ROS. These ROS can initiate lipid peroxidation within cellular membranes, producing highly reactive secondary aldehydes, such as 4-hydroxynonenal and malondialdehyde [13]. These molecules, like acetaldehyde itself, are powerful electrophiles capable of forming additional DNA adducts. Cells normally counteract such insults through antioxidant defenses, particularly glutathione, which neutralize ROS and detoxify reactive aldehydes [43]. Chronic alcohol exposure, however, can deplete glutathione reserves and weaken antioxidant capacity, enabling oxidative damage to accumulate. The resulting generation and accumulation of DNA-reactive molecules generated during alcohol metabolism and oxidative stress [28] can enhance alcohol-related cancer risk.

Alcohol metabolism can also disrupt epigenetic regulation. By interfering with one-carbon metabolism, ethanol can reduce levels of S-adenosylmethionine (the primary methyl donor for DNA methylation) and lead to activation of genes involved in cell proliferation and tumor development [44]. In addition, ethanol metabolism generates acetate that can promote histone acetylation and chromatin remodeling [45]. These epigenetic alterations may persist after alcohol exposure, leaving molecular imprints that could increase susceptibility to cancer.

Beyond its direct genotoxic effects, alcohol consumption can also influence cancer risk through alterations in the gut microbiota. Ethanol and acetaldehyde disrupt microbial community structure, promoting dysbiosis characterized by expansion of acetaldehyde-producing microbial species and loss of protective commensal organisms [46]. Ethanol and acetaldehyde can also compromise intestinal barrier function by disrupting tight junction proteins, leading to increased paracellular permeability [47]. This dysbiosis can increase local acetaldehyde production, compromise epithelial barrier integrity, and facilitate microbial translocation, leading to chronic inflammation and conditions that can promote tumor development [46]. Across these mechanisms, the strength of evidence varies. The carcinogenic role of acetaldehyde is supported by human genetic and epidemiological data on *ALDH2* and *ADH* variants [14,30,32]. However, oxidative-stress, epigenetic, and microbiota pathways have been characterized predominantly in animal and in vitro models and require additional epidemiological work [25,44,46].

Collectively, these processes can serve as drivers for alcohol-related carcinogenesis. The biological consequences of ethanol metabolism are strongly influenced by enzymatic activity, DNA repair capacity, and immune responses, all of which can be modified by environmental and lifestyle factors. This complexity highlights an important point: even though many of the molecular pathways linking alcohol to cancer are increasingly well characterized, the question of why these biochemical injuries translate into malignancy in some tissues and individuals but not in others remains.

### Why are some tissues more vulnerable than others?

The molecular injuries produced by ethanol metabolism do not affect all tissues equally. Alcohol’s carcinogenic effects, therefore, extend beyond individual cells to the tissue microenvironment, where chronic injury, inflammation, and metabolic disruption can create conditions favorable for tumor development.

Vulnerability also depends on where acetaldehyde is generated. In the oral cavity and esophagus, resident bacteria and yeasts express ADH enzymes that oxidize ethanol to acetaldehyde directly at the mucosal surface. Unlike the liver, these tissues have relatively little capacity to detoxify it, enabling acetaldehyde to accumulate in saliva at mutagenic concentrations after drinking [20,48,49]. Local exposure can be further amplified by poor oral hygiene, smoking, and ALDH2 deficiency [14]. This may help explain why the upper aerodigestive tract is disproportionately affected, relative to other tissues. What remains unclear, however, is how much of the acetaldehyde burden at a given epithelium is microbial-derived rather than host-derived, and whether local exposure is sufficient to explain tissue-specific risk (particularly at sites distant from ethanol’s point of contact).

One of the most important interfaces for alcohol toxicity is the gut–liver axis [50]. By increasing the permeability of the intestinal barrier, ethanol consumption can allow microbial products (including endotoxins and microbially produced acetaldehyde) to enter the portal circulation and reach the liver. In the liver, these molecules can activate Kupffer cells and trigger pro-inflammatory signaling pathways that release cytokines, such as TNF and IL-6 [51]. These signals generate oxidative stress and promote repeated cycles of hepatocyte injury and regeneration [52]. Over time, these cycles of cell death and proliferation create conditions in which genetic mutations can accumulate and transformed cells can expand. Chronic inflammation can also drive fibrosis, as activated hepatic stellate cells deposit extracellular matrix proteins, such as collagen, and their progressive accumulation constitutes the fibrotic scar [53]. Fibrotic remodeling alters mechanical signaling and reduces oxygen diffusion within tissues, producing localized hypoxia. Hypoxia stabilizes hypoxia-inducible factors, which promote angiogenesis and metabolic reprogramming [54]. Together, these processes reshape the tissue milieu into a tumor-promoting microenvironment. Similar processes occur in other alcohol-exposed tissues (such as the pancreas and esophagus), in which chronic oxidative stress and inflammation drive repeated cycles of tissue injury and repair [48].

Changes in hormone signaling can also contribute to tissue-specific vulnerability. Alcohol increases the activity of aromatase (which converts androgens into estrogens) [55]. Increased concentrations of estrogen stimulate proliferation of estrogen-receptor-positive cells in breast tissue, helping explain the well-established link between alcohol consumption and breast cancer risk [56]. In other tissues, metabolic disruptions may have a more important role. For example, by interfering with folate metabolism, alcohol can impair DNA synthesis and repair in rapidly dividing cells (such as those in the colon). In addition, reduced folate availability can cause uracil to be mis-incorporated into DNA, leading to increased strand breaks [57], genomic instability, and increased mutational burden, thereby facilitating tumor initiation and progression [58].

Immune surveillance can also be weakened by alcohol, an effect that may be compounded by other lifestyle factors [59,60]. Cytotoxic T cells and natural killer cells normally eliminate emerging tumor cells; chronic alcohol consumption reduces the activity of these immune cell populations [61]. As immune monitoring declines, pre-malignant clones are more likely to escape detection and expand, ultimately facilitating tumor initiation and progression.

Through these mechanisms (i.e., barrier disruption, inflammation, fibrosis, hormonal modulation, metabolic stress, immune suppression), alcohol can modify tissue microenvironments, rendering organs more susceptible to cancer. Here too, the evidence is uneven: the alcohol–estrogen pathway in breast tissue is supported by human data [56], whereas evidence for the gut–liver axis, microbiota-mediated, and immune-related mechanisms rely largely on animal and in vitro models [50,61,62]. Notably, these tissue-specific vulnerabilities rarely arise from alcohol exposure alone but instead emerge from interactions between ethanol and a broader network of co-exposures within the exposome, including diet, tobacco use, microbiota-related processes, and environmental chemicals.

## Can the exposome explain alcohol’s hidden synergies?

At the organismal level, the human body rarely encounters ethanol in isolation. Instead, individuals are simultaneously exposed to a wide range of exposome factors (e.g., environmental chemicals, dietary factors, microbial metabolites, and social influences) that can modify or interact with biological pathways affected by alcohol and/or its metabolites to promote cancer (Fig 3). For example, PFAS compounds disrupt lipid metabolism and glutathione homeostasis [22]. Indeed, combined exposure to PFAS and alcohol can exacerbate liver injury, inflammation, and metabolic dysfunction beyond what is caused by alcohol alone [22,63,64]. Similarly, many pesticides and industrial solvents undergo metabolic activation through the cytochrome P450 enzymes. Because alcohol induces CYP2E1 expression, it can accelerate the conversion of these chemicals into reactive intermediates and increase their toxicity [22]. Transition metals (e.g., iron and copper) provide another example of interaction. These metals catalyze Fenton reactions that generate highly reactive hydroxyl radicals. When combined with alcohol-induced oxidative stress, such reactions amplify cellular damage [62].

**Fig 3:**
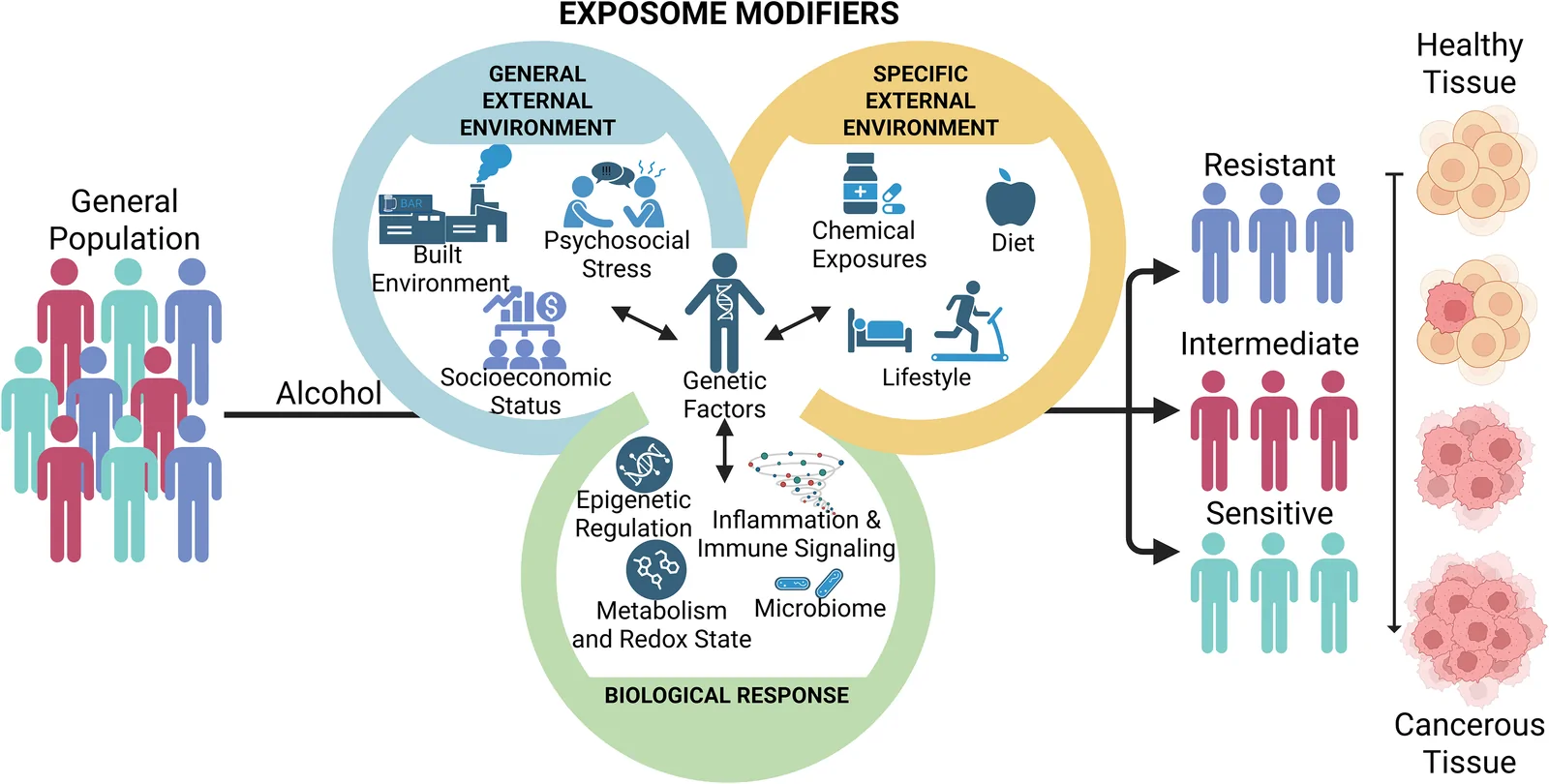
The exposome modifies individual cancer risk from a given level of alcohol exposure. For a given level of alcohol consumption, the biological responses in an individual can be influenced by a variety of factors experienced through their life, spanning general/specific external environments and internal biological factors (representative examples in each of these categories are provided). These can interact (positively or negatively, depending on the factor) with ethanol by modulating its metabolism and/or pathogenic pathways. The end result of these interactions can swing the pendulum of outcomes from minimal biological impact (e.g., healthy) to progressive tissue injury and disease (e.g., cancer). This framework illustrates why alcohol-related cancer risk varies across individuals and populations, even for similar levels of alcohol consumption. Created with Biorender.com.

Tobacco smoke represents an especially important co-exposure [65]. Many carcinogens present in tobacco require metabolic activation through pathways also used in alcohol metabolism. In addition, alcohol can facilitate carcinogenesis by acting as a solvent for tobacco-associated carcinogens [66,67]. Notably, combined exposure to alcohol and tobacco substantially increases the risk of cancers of the oral cavity and upper aerodigestive tract [68]. Dietary consumption provides additional sources of interaction. For example, nitrosamines in processed foods can interact with ethanol-derived aldehydes to form complex DNA lesions, while low folate intake can exacerbate alcohol-induced impairments in DNA synthesis and repair, together increasing mutational burden and cancer risk [69].

Beyond chemical exposures, the exposome also includes social and behavioral determinants of health [70]. Socioeconomic factors shape diet, occupational exposures to chemicals and pollutants, and patterns of alcohol consumption [71]. Chronic psychosocial stress can alter hormonal signaling and immune function [72], thereby modifying biological responses to alcohol and influencing cancer susceptibility. Biological co-exposures are also relevant, particularly for HCC [12]. As noted, chronic infection with hepatitis B or C virus interacts synergistically with alcohol to increase liver cancer risk beyond either exposure alone, underscoring that the carcinogenic impact of alcohol cannot be fully understood in isolation.

When taken together, these examples illustrate that alcohol does not act as a single, isolated carcinogenic exposure, but rather as a modifier of biological responses to a wide range of co-exposures. By altering metabolic pathways, redox balance, and detoxification capacity, alcohol can amplify the toxicity of other environmental and endogenous compounds. This interaction-based perspective may help explain why similar levels of alcohol consumption can lead to markedly different cancer risks depending on an individual’s broader exposure profile. Co-occurring exposures that independently influence cancer risk, together with differences in host genetics and underlying health status, are also likely to contribute. Importantly, the strength of evidence for these co-exposure interactions varies. The combined effects of alcohol and tobacco are supported by a substantial body of epidemiological and mechanistic evidence [11,68], including well-characterized gene–environment interactions involving *ALDH2* and the *ADH* gene cluster [30,32]. For other environmental exposures, including PFAS, pesticides, and metals, the available evidence is more heterogeneous and comes from a combination of experimental and observational studies [22,23,63,64].

Despite growing recognition of these interactions, a fundamental challenge remains: how can the combined biological effects of alcohol and co-exposures be systematically captured and quantified in real-world settings? Traditional approaches, which typically examine single exposures in isolation, are insufficient to resolve this complexity. Addressing this gap requires integrative strategies capable of simultaneously characterizing multiple exposures and their downstream biological consequences.

## How can integrative science address this question?

The chemical interactions described above highlight a central challenge in modern toxicology: most research focuses on single exposures whereas, in reality, individuals experience complex mixtures over their lives. The exposome framework addresses this limitation by considering the totality of exposures and the biological responses they generate [21]. Understanding how alcohol contributes to cancer, therefore, requires integrative approaches capable of capturing this real-world complexity.

Recent advances in exposomic technologies, particularly high-resolution mass spectrometry-based approaches such as metabolomics, lipidomics, proteomics, adductomics (measures of DNA adducts), imaging mass spectrometry, integrated multi-omics analyses, and other high-throughput assays (e.g., affinity-based proteomics), now enable comprehensive characterization of environmental exposures and their biological effects [73]. These approaches enable simultaneous measurement of thousands of small molecules, chemical adducts, and molecular signatures in human tissues and biofluids, providing an unprecedented view of the internal chemical environment. By integrating these data across biological scales—from molecular and cellular processes to tissues, organs, and whole organisms—researchers can map how alcohol interacts with co-exposures (including tobacco smoke, dietary components, environmental pollutants, and microbial metabolites) to influence key processes such as DNA damage, epigenetic regulation, inflammation, and metabolic reprogramming.

Advances in computational biology, including statistical learning, machine learning, and systems modeling, further enable the integration of multidimensional exposure and biological datasets with clinical outcomes [21]. Together, these approaches enable the identification of molecular signatures and pathways linking combined exposures to cancer-relevant phenotypes. In so doing, they provide a framework to facilitate understanding of inter-individual variability in susceptibility and identification of early biomarkers of risk. The scale at which such approaches can be applied is also expanding. Large population biobanks increasingly pair multi-omic and genotype data with electronic health record and cancer registry data, enabling features associated with cancer risk to be more accurately identified [31,73]. By encompassing participants with diverse genetic ancestries and environmental exposures, these resources offer a means to investigate how host genetics and the exposome interact with alcohol to produce different levels of cancer risk across populations.

## What will it take to divulge alcohol-related cancer risk?

Despite decades of epidemiological and mechanistic research, fundamental questions remain about how alcohol contributes to cancer development in tissues and individuals. Addressing this challenge requires moving beyond reductionist, single-exposure models toward integrative frameworks that capture the complexity of real-world biology. Recent international efforts, including dedicated scientific meetings on alcohol and cancer, have converged on the need for such approaches, emphasizing the importance of studying alcohol within the broader context of the exposome. In this framework, alcohol is not simply a carcinogen; it is a systemic modifier of biological responses that can reshape redox balance, DNA repair capacity, immune function, and metabolic pathways. Through these effects, alcohol both influences and is influenced by co-occurring exposures across the exposome. A central challenge is that alcohol operates within a network of interacting biological and environmental factors. Genetic variation in alcohol-metabolizing enzymes and DNA repair pathways can influence susceptibility, while co-exposures, including tobacco smoke, environmental pollutants, dietary factors, and microbial metabolism, represent only a subset of the broader exposome shaping alcohol-related carcinogenesis, much of which remains uncharacterized and requires discovery-driven investigation. Therefore, understanding alcohol-related carcinogenesis requires studying alcohol as part of a dynamic exposure system rather than as an isolated agent. Advances in high-resolution mass spectrometry-based approaches, together with powerful computational modeling, now provide the tools to map these interactions across scales—from molecular damage to population-level risk—and, within a translational exposomics framework, to integrate the exposome with genomic and other omics data to better understand how environmental exposures and host susceptibility jointly influence cancer risk [21].

Framed in this way, several key questions emerge (Box 1): which acetaldehyde-derived lesions drive mutagenesis in human tissues [27,33,34]; how these lesions generate the mutational signatures observed in alcohol-associated tumors [38]; how co-exposures and host genetics shape cancer risk across populations; and to what extent alcohol-induced molecular changes are reversible following reduced consumption or abstinence. Epidemiological evidence indicates that reducing or stopping alcohol consumption lowers cancer risk, most consistently for cancers of the oral cavity and esophagus, although the magnitude and timing of risk reduction remain poorly defined [74]. Cessation is expected to reverse acetaldehyde-driven genotoxicity, oxidative stress, and gut barrier dysfunction, yet the timing and extent of these changes have not been systematically characterized in humans. Advances in DNA adductomics [28], mutational-signature analysis [38], longitudinal exposomic profiling, and large multi-omic biobanks now provide complementary approaches for directly linking alcohol exposure to molecular damage, characterizing recovery trajectories, identifying biomarkers of reversible and persistent cancer risk, and defining how environmental exposures and host genetics jointly influence cancer risk [31,73].

**Box 1. Key open questions in alcohol–cancer research and potential approaches to solving them.**

**Which molecular lesions are most responsible for alcohol-induced carcinogenesis?**

Acetaldehyde forms DNA adducts and crosslinks, but ethanol metabolism also generates reactive oxygen species and lipid peroxidation products. Distinguishing the lesions that drive the mutational signatures of alcohol-associated cancers from those that simply accompany exposure needs to be better delineated.

*Basic science:* DNA adductomics approaches that directly quantify acetaldehyde-derived adducts in exposed tissues, combined with functional repair deficiency models, could help establish which molecular lesions are causally linked to mutagenesis rather than merely correlated with exposure.

*Population science:* The linking of human biomonitoring data on DNA adduct burden to tumor mutational signature analyses in large sequenced cohorts could clarify which molecular lesion types underlie the mutational signatures observed in alcohol-associated tumors.

**How do DNA repair pathways determine individual susceptibility?**

Several DNA repair pathways help resolve alcohol-induced DNA damage. How variations in these pathways contribute to alcohol-induced cancer risk in individuals remains poorly understood.

*Basic science:* Mouse models combining alcohol exposure with targeted deficiencies in Fanconi anemia, nucleotide excision, or mismatch repair genes can be used to investigate how specific repair pathways modify cancer risk.

*Population science:* Genome-wide association and exome studies stratifying alcohol-related cancer risk by DNA repair gene variants in large biobank cohorts could identify high-risk genetic subgroups.

**What is the role of the microbiome in alcohol-related carcinogenesis?**

Oral and gut microbes can convert ethanol to acetaldehyde, potentially generating carcinogenic concentrations directly at epithelial surfaces. How microbial metabolism contributes to cancer risk in different tissues remains an active area of investigation.

*Basic science:* Gnotobiotic or germ-free animal models colonized with defined acetaldehyde-producing (versus acetaldehyde non-producing bacterial strains) would help establish whether microbial acetaldehyde production is causally sufficient to drive local carcinogenesis.

*Population science:* Prospective cohort studies pairing oral and gut microbiome profiling with alcohol consumption and precancerous lesion surveillance could clarify the contribution of the microbiome to human cancer risk.

**Why are some tissues particularly vulnerable to alcohol-induced cancer?**

Cancers associated with alcohol arise primarily in the upper aerodigestive tract, liver, colorectum, and breast. Differences in metabolism, local acetaldehyde exposure, hormone regulation, and tissue-specific DNA repair capacity may all contribute to the observed variability.

*Basic science:* Comparative multi-tissue profiling of alcohol-metabolizing enzyme expression and DNA repair capacity across organs in animal models could help explain differential tissue susceptibility.

*Population science:* Cancer registry analyses comparing alcohol dose–response relationships across cancer tissue sites within the same populations could reveal which biomarkers best predict site-specific risk.

**How do environmental co-exposures amplify alcohol’s carcinogenic effects?**

Alcohol frequently co-occurs with other exposures, such as tobacco smoke, environmental pollutants, metals, solvents, and dietary carcinogens. Understanding how these mixtures interact biologically is essential for explaining real-world cancer risk.

*Basic science:* Controlled co-exposure studies in animal models can help establish causal interactions and underlying mechanisms.

*Population science:* Exposome-wide association studies linking biomonitoring data with alcohol consumption and cancer incidence in large cohorts could facilitate identification of which co-exposures matter most for real-world risk.

**Can exposomic and multi-omic approaches reveal early biomarkers of alcohol-related carcinogenesis?**

Emerging technologies, particularly high-resolution mass spectrometry-based approaches, may help to identify early molecular signatures of alcohol exposure and cancer susceptibility.

*Basic science:* Longitudinal multi-omic profiling in animal models (from initial exposure through tumor development) could elucidate early molecular signatures with predictive value.

*Population science:* Prospective biobank studies with linked proteomic/metabolomic data and long-term clinical follow-up could validate candidate biomarkers against real-world cancer incidence.

The proposed integrative approach bridges traditionally siloed disciplines (e.g., toxicology, molecular biology, epidemiology, microbiome science, and environmental health) and offers a path toward resolving the long-standing variability and uncertainty that characterize alcohol-related cancer risk. Ultimately, embracing a translational exposomics perspective will not only deepen mechanistic understanding but also enable more precise prevention strategies by accounting for the cumulative and interacting exposures that drive cancer risk over the life course.

As we learn more about the processes by which alcohol promotes cancer, it remains important to recognize that alcohol increases cancer risk; its consumption represents a preventable public health risk.

## Abbreviations

ADH: alcohol dehydrogenase
HCC: hepatocellular carcinoma
ROS: reactive oxygen species

## References

[1] Ferrari P, Rumgay H, Weiderpass E, Soerjomataram I. Alcohol and cancer: The epidemiological evidence. Alcohol and alcohol-related diseases. Springer International Publishing; 2023. p. 1371–89. doi: 10.1007/978-3-031-32483-3_74

[2] Calonge BN, Stone KB. Review of evidence on alcohol and health. Washington (DC): National Academies Press; 2025. 40393408

[3] Jin J, Zhang Y, Huang Y. The relationship between tobacco and non-alcoholic fatty liver disease incidence: A systematic review and meta-analysis of observational studies. Front Med (Lausanne). 2025;12:1670932. doi: 10.3389/fmed.2025.1670932 41169417 PMC12568600

[4] Nath P, Anand AC. Extrahepatic manifestations in alcoholic liver disease. J Clin Exp Hepatol. 2022;12(5):1371–83. doi: 10.1016/j.jceh.2022.02.004 36157144 PMC9499846

[5] Fink H, Langselius O, Vignat J, Rumgay H, Rehm J, Martinez RX, et al. Global and regional cancer burden attributable to modifiable risk factors to inform prevention. Nat Med. 2026;32(4):1306–15. doi: 10.1038/s41591-026-04219-7 41634393

[6] Islami F, Marlow EC, Thomson B, McCullough ML, Rumgay H, Gapstur SM, et al. Proportion and number of cancer cases and deaths attributable to potentially modifiable risk factors in the United States, 2019. CA Cancer J Clin. 2024;74(5):405–32. doi: 10.3322/caac.21858 38990124

[7] IARC Working Group on the Evaluation of Carcinogenic Risks to Humans. Personal habits and indoor combustions. IARC Monogr Eval Carcinog Risks Hum. 2012;100(Pt E):1–538. 23193840 PMC4781577

[8] Gapstur SM, Bandera EV, Jernigan DH, LoConte NK, Southwell BG, Vasiliou V, et al. Alcohol and cancer: Existing knowledge and evidence gaps across the cancer continuum. Cancer Epidemiol Biomarkers Prev. 2022;31(1):5–10. doi: 10.1158/1055-9965.EPI-21-0934 34728469 PMC8755600

[9] Bagnardi V, Rota M, Botteri E, Tramacere I, Islami F, Fedirko V, et al. Alcohol consumption and site-specific cancer risk: A comprehensive dose-response meta-analysis. Br J Cancer. 2015;112(3):580–93. doi: 10.1038/bjc.2014.579 25422909 PMC4453639

[10] Rumgay H, Murphy N, Ferrari P, Soerjomataram I. Alcohol and cancer: Epidemiology and biological mechanisms. Nutrients. 2021;13(9):3173. doi: 10.3390/nu13093173 PMC8470184 34579050

[11] Jun S, Park H, Kim U-J, Lee HA, Park B, Lee SY, et al. The combined effects of alcohol consumption and smoking on cancer risk by exposure level: a systematic review and meta-analysis. J Korean Med Sci. 2024;39(22):e185. doi: 10.3346/jkms.2024.39.e185 38859742 PMC11164648

[12] Poorolajal J, Shadi Y, Heshmati B. Interaction between hepatitis B, hepatitis C and alcohol in the development of hepatocellular carcinoma: A systematic review and meta-analysis. J Viral Hepat. 2025;32(1):e14042. doi: 10.1111/jvh.14042 39716779

[13] Johnson CH, Golla JP, Dioletis E, Singh S, Ishii M, Charkoftaki G, et al. Molecular mechanisms of alcohol-induced colorectal carcinogenesis. Cancers (Basel). 2021;13(17):4404. doi: 10.3390/cancers13174404 34503214 PMC8431530

[14] Chen C-H, Wang W-L, Hsu M-H, Mochly-Rosen D. Alcohol consumption, ALDH2 polymorphism as risk factors for upper aerodigestive tract cancer progression and prognosis. Life (Basel). 2022;12(3):348. doi: 10.3390/life12030348 35330099 PMC8956056

[15] Rattray NJW, Charkoftaki G, Rattray Z, Hansen JE, Vasiliou V, Johnson CH. Environmental influences in the etiology of colorectal cancer: the premise of metabolomics. Curr Pharmacol Rep. 2017;3(3):114–25. doi: 10.1007/s40495-017-0088-z 28642837 PMC5475285

[16] Seol JE, Kim J, Lee B-H, Hwang D-Y, Jeong J, Lee H-J, et al. Folate, alcohol, ADH1B and ALDH2 and colorectal cancer risk. Public Health Nutr. 2021;24(4):677–84. doi: 10.1017/S136898001900452X 32223781 PMC11574830

[17] Abbas D, Ciricillo JA, Elom HA, Moon AM. Extrahepatic health effects of alcohol use and alcohol-associated liver disease. Clin Ther. 2023;45(12):1201–11. doi: 10.1016/j.clinthera.2023.08.018 37806811

[18] Katikireddi SV, Whitley E, Lewsey J, Gray L, Leyland AH. Socioeconomic status as an effect modifier of alcohol consumption and harm: analysis of linked cohort data. Lancet Public Health. 2017;2(6):e267–76. doi: 10.1016/S2468-2667(17)30078-6 28626829 PMC5463030

[19] Martin A, Thakur BK. Diet and environment in colorectal cancer development, roles of. In: Kuipers EJ, editor. Encyclopedia of gastroenterology. 2nd ed. Oxford: Academic Press; 2020. p. 33–50.

[20] Scherübl H. Alcohol use and gastrointestinal cancer risk. Visc Med. 2020;36(3):175–81. doi: 10.1159/000507232 32775347 PMC7383256

[21] Sarigiannis D, Karakitsios S, Anesti O, Stem A, Valvi D, Sumner SCJ, et al. Advancing translational exposomics: Bridging genome, exposome and personalized medicine. Hum Genomics. 2025;19(1):48. doi: 10.1186/s40246-025-00761-6 40307849 PMC12044731

[22] Stem AD, Tieghi RS, Chatzi VL, Kleinstreuer N, Valvi D, Thompson DC, et al. Synergistic toxicity in alcohol-associated liver disease and PFAS exposure. Toxicol Sci. 2025;208(1):9–31. doi: 10.1093/toxsci/kfaf110 40737496 PMC12599875

[23] Rocha JBTd, Aschner M, Nogara PA, Virgolini MB. Toxicology of essential and xenobiotic metals. 2024. 97 p.

[24] Favoretto CA, Nunes YC, Macedo GC, Lopes JSR, Quadros IMH. Chronic social defeat stress: Impacts on ethanol-induced stimulation, corticosterone response, and brain monoamine levels. J Psychopharmacol. 2020;34(4):412–9. doi: 10.1177/0269881119900983 31965898

[25] Zakhari S, Neuman M, Seitz HK. The role of cytochrome P4502E1 in ethanol mediated diseases: A narrative update. Alcohol Alcohol. 2025;60(3):agaf014. doi: 10.1093/alcalc/agaf014 40192654

[26] Seitz HK. The role of cytochrome P4502E1 in the pathogenesis of alcoholic liver disease and carcinogenesis. Chem Biol Interact. 2020;316:108918. doi: 10.1016/j.cbi.2019.108918 31836462

[27] Brooks PJ, Theruvathu JA. DNA adducts from acetaldehyde: Implications for alcohol-related carcinogenesis. Alcohol. 2005;35(3):187–93. doi: 10.1016/j.alcohol.2005.03.009 16054980

[28] Guidolin V, Carlson ES, Carrà A, Villalta PW, Maertens LA, Hecht SS. Identification of new markers of alcohol-derived DNA damage in humans. Biomolecules. 2021;11(3):366. doi: 10.3390/biom11030366 PMC7997542 33673538

[29] Rwere F, White JR, Hell RCR, Yu X, Zeng X, McNeil L, et al. Uncovering newly identified aldehyde dehydrogenase 2 genetic variants that lead to acetaldehyde accumulation after an alcohol challenge. J Transl Med. 2024;22(1):697. doi: 10.1186/s12967-024-05507-x 39075523 PMC11288122

[30] Wu C, Hu Z, He Z, Jia W, Wang F, Zhou Y, et al. Genome-wide association study identifies three new susceptibility loci for esophageal squamous-cell carcinoma in Chinese populations. Nat Genet. 2011;43(7):679–84. doi: 10.1038/ng.849 21642993

[31] Ghouse J, Sveinbjörnsson G, Vujkovic M, Seidelin A-S, Gellert-Kristensen H, Ahlberg G, et al. Integrative common and rare variant analyses provide insights into the genetic architecture of liver cirrhosis. Nat Genet. 2024;56(5):827–37. doi: 10.1038/s41588-024-01720-y 38632349 PMC11096111

[32] Wu C, Kraft P, Zhai K, Chang J, Wang Z, Li Y, et al. Genome-wide association analyses of esophageal squamous cell carcinoma in Chinese identify multiple susceptibility loci and gene-environment interactions. Nat Genet. 2012;44(10):1090–7. doi: 10.1038/ng.2411 22960999

[33] Hodskinson MR, Bolner A, Sato K, Kamimae-Lanning AN, Rooijers K, Witte M, et al. Alcohol-derived DNA crosslinks are repaired by two distinct mechanisms. Nature. 2020;579(7800):603–8. doi: 10.1038/s41586-020-2059-5 32132710 PMC7116288

[34] Garaycoechea JI, Crossan GP, Langevin F, Mulderrig L, Louzada S, Yang F, et al. Alcohol and endogenous aldehydes damage chromosomes and mutate stem cells. Nature. 2018;553(7687):171–7. doi: 10.1038/nature25154 29323295 PMC6047743

[35] Peake JD, Noguchi C, Lin B, Theriault A, O’Connor M, Sheth S, et al. FANCD2 limits acetaldehyde-induced genomic instability during DNA replication in esophageal keratinocytes. Mol Oncol. 2021;15(11):3109–24. doi: 10.1002/1878-0261.13072 34328261 PMC8564632

[36] Subramaiyam N. Insights of mitochondrial involvement in alcoholic fatty liver disease. J Cell Physiol. 2023;238(10):2175–90. doi: 10.1002/jcp.31100 37642259

[37] Tan HK, Yates E, Lilly K, Dhanda AD. Oxidative stress in alcohol-related liver disease. World J Hepatol. 2020;12(7):332–49. doi: 10.4254/wjh.v12.i7.332 32821333 PMC7407918

[38] Vijayraghavan S, Porcher L, Mieczkowski PA, Saini N. Acetaldehyde makes a distinct mutation signature in single-stranded DNA. Nucleic Acids Res. 2022;50(13):7451–64. doi: 10.1093/nar/gkac570 35776120 PMC9303387

[39] Yamazaki K, Iguchi T, Kanoh Y, Takayasu K, Ngo TTT, Onuki A, et al. Homologous recombination contributes to the repair of acetaldehyde-induced DNA damage. Cell Cycle. 2024;23(4):369–84. doi: 10.1080/15384101.2024.2335028 38571319 PMC11174073

[40] Cerretelli G, Zhou Y, Müller MF, Adams DJ, Arends MJ. Acetaldehyde and defective mismatch repair increase colonic tumours in a Lynch syndrome model with Aldh1b1 inactivation. Dis Model Mech. 2023;16(8):dmm050240. doi: 10.1242/dmm.050240 37395714 PMC10417510

[41] Sonohara Y, Takatsuka R, Masutani C, Iwai S, Kuraoka I. Acetaldehyde induces NER repairable mutagenic DNA lesions. Carcinogenesis. 2022;43(1):52–9. doi: 10.1093/carcin/bgab087 34546339

[42] Charkoftaki G, Tan WY, Berrios-Carcamo P, Orlicky DJ, Golla JP, Garcia-Milian R, et al. Liver metabolomics identifies bile acid profile changes at early stages of alcoholic liver disease in mice. Chem Biol Interact. 2022;360:109931. doi: 10.1016/j.cbi.2022.109931 35429548 PMC9364420

[43] Ryu T, Kim K, Choi SE, Chung KPS, Jeong W-I. New insights in the pathogenesis of alcohol-related liver disease: The metabolic, immunologic, and neurologic pathways. Liver Res. 2022;7(1):1–8. doi: 10.1016/j.livres.2022.09.004 39959703 PMC11791844

[44] Zakhari S. Alcohol metabolism and epigenetics changes. Alcohol Res. 2013;35(1):6–16. doi: 10.35946/arcr.v35.1.02 24313160 PMC3860421

[45] Pandey SC, Bohnsack JP. Alcohol makes its epigenetic marks. Cell Metab. 2020;31(2):213–4. doi: 10.1016/j.cmet.2020.01.008 PMC7162615 32023443

[46] Schöler D, Schnabl B. The role of the microbiome in liver disease. Curr Opin Gastroenterol. 2024;40(3):134–42. doi: 10.1097/MOG.0000000000001013 38362864 PMC10990783

[47] Meena AS, Shukla PK, Bell B, Giorgianni F, Caires R, Fernández-Peña C, et al. TRPV6 channel mediates alcohol-induced gut barrier dysfunction and systemic response. Cell Rep. 2022;39(11):110937. doi: 10.1016/j.celrep.2022.110937 35705057 PMC9250449

[48] Seitz HK, Stickel F. Molecular mechanisms of alcohol-mediated carcinogenesis. Nat Rev Cancer. 2007;7(8):599–612. doi: 10.1038/nrc2191 17646865

[49] Nieminen MT, Salaspuro M. Local Acetaldehyde-An Essential Role in Alcohol-Related Upper Gastrointestinal Tract Carcinogenesis. Cancers (Basel). 2018;10(1):11. doi: 10.3390/cancers10010011 29303995 PMC5789361

[50] Raya Tonetti F, Eguileor A, Mrdjen M, Pathak V, Travers J, Nagy LE, et al. Gut-liver axis: Recent concepts in pathophysiology in alcohol-associated liver disease. Hepatology. 2024;80(6):1342–71. doi: 10.1097/HEP.0000000000000924 38691396 PMC11801230

[51] Thakur V, McMullen MR, Pritchard MT, Nagy LE. Regulation of macrophage activation in alcoholic liver disease. J Gastroenterol Hepatol. 2007;22 Suppl 1:S53-6. doi: 10.1111/j.1440-1746.2006.04650.x 17567466

[52] Szabo G, Petrasek J. Gut-liver axis and sterile signals in the development of alcoholic liver disease. Alcohol Alcohol. 2017;52(4):414–24. doi: 10.1093/alcalc/agx025 28482064 PMC5860369

[53] Slevin E, Baiocchi L, Wu N, Ekser B, Sato K, Lin E, et al. Kupffer Cells: Inflammation Pathways and Cell-Cell Interactions in Alcohol-Associated Liver Disease. Am J Pathol. 2020;190(11):2185–93. doi: 10.1016/j.ajpath.2020.08.014 32919978 PMC7587925

[54] Foglia B, Novo E, Protopapa F, Maggiora M, Bocca C, Cannito S, et al. Hypoxia, hypoxia-inducible factors and liver fibrosis. Cells. 2021;10(7):1764. doi: 10.3390/cells10071764 PMC8305108 34359934

[55] Finn DA. The Endocrine System and Alcohol Drinking in Females. Alcohol Research. 2020;40(2):02. doi: 10.35946/arcr.v40.2.02 PMC7374925 32714716

[56] Tin Tin S, Smith-Byrne K, Ferrari P, Rinaldi S, McCullough ML, Teras LR, et al. Alcohol intake and endogenous sex hormones in women: Meta-analysis of cohort studies and Mendelian randomization. Cancer. 2024;130(19):3375–86. doi: 10.1002/cncr.35391 38824654

[57] Sharma J, Krupenko SA. Folate pathways mediating the effects of ethanol in tumorigenesis. Chem Biol Interact. 2020;324:109091. doi: 10.1016/j.cbi.2020.109091 32283069 PMC7232643

[58] Zakhari S, Hoek JB. Epidemiology of moderate alcohol consumption and breast cancer: association or causation?. Cancers (Basel). 2018;10(10):agaf014. doi: 10.3390/cancers10100349 PMC6210419 30249004

[59] Kim A, Bellar A, McMullen MR, Li X, Nagy LE. Functionally Diverse Inflammatory Responses in Peripheral and Liver Monocytes in Alcohol-Associated Hepatitis. Hepatol Commun. 2020;4(10):1459–76. doi: 10.1002/hep4.1563 33024916 PMC7527760

[60] Szabo G. Consequences of alcohol consumption on host defence. Alcohol Alcohol. 1999;34(6):830–41. doi: 10.1093/alcalc/34.6.830 10659718

[61] Molina JC, Guerrero-Morán JD, González-Espinosa C. Alcohol: Immunomodulatory Effects and Cancer. Rev Invest Clin. 2023;75(3):129–42. doi: 10.24875/RIC.23000116 37441764

[62] Chen Y, Gao W, Cai K, Yang L, Chu H. Ferroptosis and gut microbiota: A new horizon in alcohol-associated liver disease management. Cell Mol Life Sci. 2025;82(1):282. doi: 10.1007/s00018-025-05815-5 40682668 PMC12276196

[63] Ekuban FA, Gripshover TC, Ames P, Biswas K, Bolatimi OE, Abramson J, et al. Perfluorooctane sulfonate exposure and alcohol-associated liver disease severity in a mouse chronic-binge ethanol feeding model. Toxicol Sci. 2025;206(2):388–408. doi: 10.1093/toxsci/kfaf066 40347464 PMC12329793

[64] Stem AD, Alayyoub M, Aalizadeh R, Nikolopoulou V, Lisgara A, Shvartsman A. Integrated multi-omics reveals synergistic hepatotoxicity of ethanol and PFOS co-exposure. Chem Biol Interact. 2026;434:112101. doi: 10.1016/j.cbi.2026.112101 42036057

[65] Hecht SS, Stepanov I, Carmella SG. Exposure and metabolic activation biomarkers of carcinogenic tobacco-specific nitrosamines. Acc Chem Res. 2016;49(1):106–14. doi: 10.1021/acs.accounts.5b00472 26678241 PMC5154679

[66] IARC Working Group on the Evaluation of Carcinogenic Risks to Humans. Alcohol consumption and ethyl carbamate. IARC monographs on the evaluation of carcinogenic risks to humans. 2010;96:3-1383. PMC4781168 21735939

[67] Saracci R. The interactions of tobacco smoking and other agents in cancer etiology. Epidemiol Rev. 1987;9:175–93. doi: 10.1093/oxfordjournals.epirev.a036301 3315716

[68] Canova C, Richiardi L, Merletti F, Pentenero M, Gervasio C, Tanturri G, et al. Alcohol, tobacco and genetic susceptibility in relation to cancers of the upper aerodigestive tract in northern Italy. Tumori. 2010;96(1):1–10. doi: 10.1177/030089161009600101 20437850

[69] Chetwood JD, Garg P, Finch P, Gordon M. Systematic review: The etiology of esophageal squamous cell carcinoma in low-income settings. Expert Rev Gastroenterol Hepatol. 2019;13(1):71–88. doi: 10.1080/17474124.2019.1543024 30791842

[70] Daly MC, Duncan GJ, McDonough P, Williams DR. Optimal indicators of socioeconomic status for health research. Am J Public Health. 2002;92(7):1151–7. doi: 10.2105/ajph.92.7.1151 12084700 PMC1447206

[71] Di Renzo L, Gualtieri P, Frank G, Cianci R, Caldarelli M, Leggeri G, et al. Exploring the exposome spectrum: Unveiling endogenous and exogenous factors in non-communicable chronic diseases. Diseases. 2024;12(8):176. doi: 10.3390/diseases12080176 39195175 PMC11353379

[72] Lawrence WR, McDonald JA, Williams F, Shiels MS, Freedman ND, Lin Z, et al. Stressful life events, social support, and incident breast cancer by estrogen receptor status. Cancer Prev Res (Phila). 2023;16(5):259–67. doi: 10.1158/1940-6207.CAPR-22-0472 37067915 PMC10159918

[73] Papier K, Atkins JR, Tong TYN, Gaitskell K, Desai T, Ogamba CF, et al. Identifying proteomic risk factors for cancer using prospective and exome analyses of 1463 circulating proteins and risk of 19 cancers in the UK Biobank. Nat Commun. 2024;15(1):4010. doi: 10.1038/s41467-024-48017-6 38750076 PMC11096312

[74] Gapstur SM, Bouvard V, Nethan ST, Freudenheim JL, Abnet CC, English DR, et al. The IARC perspective on alcohol reduction or cessation and cancer risk. N Engl J Med. 2023;389(26):2486–94. doi: 10.1056/NEJMsr2306723 38157507

